# Wegener's Granulomatosis with Extensive Bone Abnormalities Mimicking Fungal Sinusitis

**DOI:** 10.1155/2012/103403

**Published:** 2012-06-26

**Authors:** Satoru Kodama, Nozomi Nomi, Masashi Suzuki

**Affiliations:** Department of Otolaryngology, Oita University Faculty of Medicine, 1-1 Idaigaoka, Hazama-Machi, Oita, Yufu 879-5593, Japan

## Abstract

Abnormalities of the underlying bone of the paranasal sinuses have sometimes been shown in Wegener's granulomatosis (WG). We describe an interesting case of WG with extensive bone abnormalities in the sinuses mimicking fungal sinusitis. A 30-year-old woman presented with intermittent unilateral epistaxis. Biopsy was performed for the granulation tissue in the right nasal cavity, and she was diagnosed as having WG. Computed tomography (CT) revealed a ring-like calcification, mimicking a fungus ball, in the right maxillary sinus. Endoscopic sinus surgery was performed to confirm the diagnosis. A spherical bony structure, surrounded by granulation tissue, was identified in the maxillary sinus. The wall of the “bony ball” was fragile, like an egg shell. No fungus was found in the sinus. Thus, the extensive bone abnormalities were due to WG.

## 1. Introduction

Wegener's granulomatosis (WG) is a systemic vasculitis that usually involves the upper and lower respiratory tracts and kidneys. Mucosal abnormalities in the nose and paranasal sinuses have been well characterized in patients with WG. Epistaxis and crust formation are frequently shown in these patients. In addition to mucosal abnormalities, abnormalities of the underlying bone of the sinuses have recently been reported [1–4]. Herein, we describe an interesting case of WG with extensive bone abnormalities in the sinuses mimicking fungal sinusitis.

## 2. Case Presentation

A 30-year-old woman presented with intermittent right-sided epistaxis. When she was 18 years of age, both kidney and lung diseases appeared. Goodpasture's syndrome was diagnosed, and she was treated with corticosteroids for 12 years. Her renal function gradually worsened, and hemodialysis was introduced 4 months before the first episode of epistaxis. She had no history of nasal trauma or nasal surgery.

**Figure 1:**
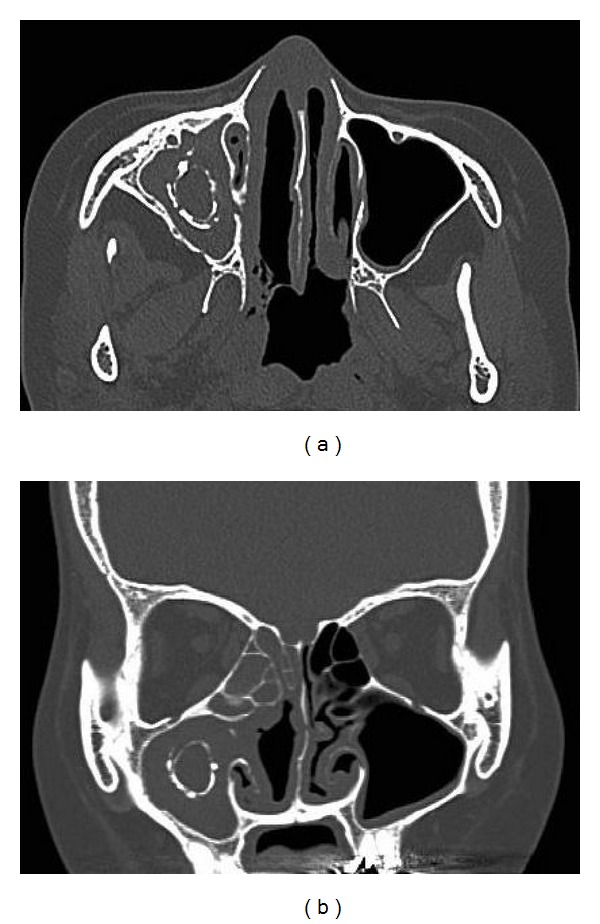
Horizontal (a) and axial (b) CT image. Ring-like calcification is seen in the right maxillary sinus.

**Figure 2:**
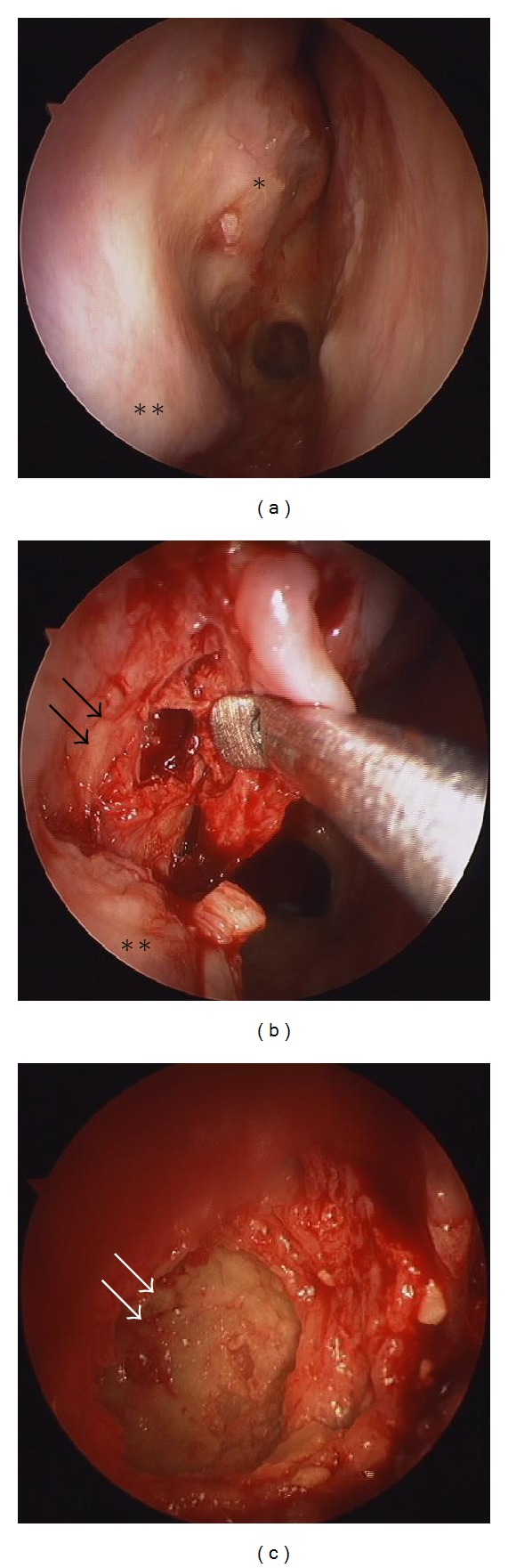
Operative findings. (a) Right nasal cavity; (b) a spherical bony structure occupies the right maxillary sinus (black arrows); (c) the inside of the bony ball. The wall is fragile, like an egg shell (white arrows); *the remnant of the middle turbinate; **inferior turbinate.

Anterior rhinoscopy revealed granulation tissue, which bled easily, in the right nasal cavity. The right middle turbinate was absent. There was no septal perforation, and the left nasal cavity was normal. The granulation tissue was biopsied, and pathologic examination revealed granulomatous vasculitis, which corresponded to WG. She had been diagnosed as Goodpasture's syndrome for a long term; however, involvement of the nose, lung, and kidney led to a final diagnosis of WG. The serum c-antineutrophil cytoplasmic antibody concentration was 68 EU. Computed tomography (CT) revealed extensive bone abnormalities in the right sinuses. Ring-like calcification, mimicking a fungus ball, was detected in the right maxillary sinus. Osteosclerotic change was also apparent in the sinus wall (Figures 1(a) and 1(b)). The right frontal sinus was filled with calcification, and opacities were visible in the right ethmoid and sphenoid sinuses. Magnetic resonance imaging was also performed; however, differential diagnosis was difficult. Whereas WG shows various radiographic changes in the sinus, a typical CT finding of fungal sinusitis shows the opacification of the sinus with calcification (fungus ball). Fungal sinusitis due to the long-term steroid therapy was thought possible. Thus, the possibility of a fungus ball had to be excluded before immunosuppressive therapy could be continued. Endoscopic sinus surgery was performed under general anesthesia to confirm the diagnosis of WG. Anatomical and surgical landmark was unclear in the right nasal cavity (Figure 2(a)). A mucosal flap was made in the middle meatus, and the maxillary sinus was opened. A spherical bony structure, surrounded by granulation tissue, was identified in the maxillary sinus (Figure 2(b)). The wall of the “bony ball” was fragile, like an egg shell, and there was serous fluid inside the ball (Figure 2(c)). No fungus ball was found in the sinus. Pathologic examination of the bony ball revealed increased collagenous tissue with calcification. No fungus was detected. Thus, the extensive bone abnormalities were due to WG. The postoperative course was uneventful, and the patient is now undergoing serial corticosteroid treatment for WG.

## 3. Discussion

The typical histopathologic lesion in WG of the respiratory tract is necrotizing vasculitis. This is accompanied by an inflammatory cell infiltrate within the vessel wall or the presence of large epithelioid granulomas with obliteration of adjacent small arteries. The resulting avascular necrosis accounts for the bone destruction, that is the, most striking change apparent on CT and is seen against a background of generalized mucosal thickening in the nose and sinuses [1, 2]. In addition to bone destruction, some patients show sclerotic changes in the walls of the affected sinuses [1–4]. This presents a distinctive appearance on CT. With bone windowing, the sclerotic change is seen as a slightly irregular line running parallel to the sinus wall, comprising a new corticated edge inside the normal bone boundary [2]. Although neo-osteogenesis is usually seen along the sinus wall, in the present case, a spherical “bony ball,” mimicking a fungus ball, was seen in the center of the maxillary sinus. The extensive bone abnormalities seen in the present case have not been reported previously in the patients with WG.

The pathophysiology of bony abnormalities in the sinuses of the patients with WG has not been elucidated. The most likely mechanism is the development of chronic periostitis from granulomatous and vasculitic involvement of the sinus mucosa and periosteum, which stimulates new bone deposition [1, 5]. Another theory suggests that chronic bacterial periostitis develops from the longstanding, severe sinusitis associated with WG [3]. The dramatic bony changes may be a result of a compounded effect of both infectious and vasculitic processes. 

Differential diagnosis of bony thickening and neo-osteogenesis in the sinuses is fairly limited. A benign tumor such as ossifying fibroma, osteoma, or fibrous dysplasia should be considered, but each of these should have characteristic radiologic features that allow for diagnosis. In the present case, clinical histories and radiologic findings mimicked those of fungal sinusitis. Although bony changes, including neo-osteogenesis, bony obliteration, and bony erosion, may provide radiologic evidence of underlying WG, changes in addition to those reported in the literature may accompany WG.
